# Development of a reverse transcription recombinase polymerase amplification assay for rapid and direct visual detection of Severe Acute Respiratory Syndrome Coronavirus 2 (SARS-CoV-2)

**DOI:** 10.1371/journal.pone.0245164

**Published:** 2021-01-06

**Authors:** Yee Ling Lau, Ilyiana binti Ismail, Nur Izati binti Mustapa, Meng Yee Lai, Tuan Suhaila Tuan Soh, Afifah Haji Hassan, Kalaiarasu M. Peariasamy, Yee Leng Lee, Maria Kahar Bador Abdul Kahar, Jennifer Chong, Pik Pin Goh

**Affiliations:** 1 Department of Parasitology, Faculty of Medicine, University of Malaya, Kuala Lumpur, Malaysia; 2 Department of Pathology, Hospital Sungai Buloh, Selangor, Malaysia; 3 Clinical Research Centre, Hospital Sungai Buloh, Selangor, Malaysia; 4 Department of Medical Microbiology, Faculty of Medicine, University of Malaya, Kuala Lumpur, Malaysia; 5 Institute for Clinical Research (ICR), National Institutes of Health (NIH), Ministry of Health Malaysia, Putrajaya, Malaysia; Waseda University, JAPAN

## Abstract

Rapid diagnosis is an important intervention in managing the Severe Acute Respiratory Syndrome Coronavirus 2 (SARS-CoV-2) outbreak. Real time reverse transcription polymerase chain reaction (RT-qPCR) remains the primary means for diagnosing the new virus strain but it is time consuming and costly. Recombinase polymerase amplification (RPA) is an isothermal amplification assay that does not require a PCR machine. It is an affordable, rapid, and simple assay. In this study, we developed and optimized a sensitive reverse transcription (RT)-RPA assay for the rapid detection of SARS-CoV-2 using SYBR Green I and/or lateral flow (LF) strip. The analytical sensitivity and specificity of the RT-RPA assay were tested by using 10-fold serial diluted synthetic RNA and genomic RNA of similar viruses, respectively. Clinical sensitivity and specificity of the RT-RPA assay were carried out using 78 positive and 35 negative nasopharyngeal samples. The detection limit of both RPA and RT-qPCR assays was 7.659 and 5 copies/μL RNA, respectively with no cross reactivity with other viruses. The clinical sensitivity and specificity of RT-RPA were 98% and 100%, respectively. Our study showed that RT-RPA represents a viable alternative to RT-qPCR for the detection of SARS-CoV-2, especially in areas with limited infrastructure.

## Introduction

The coronavirus disease 2019 (COVID-2019) outbreak was declared as a global health emergency by the World Health Organization (WHO) on 30 January 2020 [1]. Several real-time reverse transcription polymerase chain reaction (RT-qPCR) methods have been developed for the detection of SARS-like coronaviruses and the specific detection of SARS-CoV-2 immediately since the outbreak. RT-qPCR requires costly machines and consumables which are standard in a reference laboratory. However, the time required from transporting the sample to obtaining the results may need 2–3 days [2]. In a public health emergency, this time-consuming and expensive testing method is treated less favourably.

Thus, rapid and cost-effective diagnostic tools that can specifically diagnose this infection are urgently needed. Recombinase polymerase amplification (RPA) is a highly specific and sensitive isothermal amplification technique (37–42°C) that can amplify DNA/RNA as low as 1 copy per reaction in < 30 mins. RPA has successfully adopted successfully for different kinds of target organisms: virus, bacteria, protozoa, and fungi with various sample types [3]. In this study, a reverse transcription (RT) RPA assay was developed and optimized for the detection of SARS-CoV-2 in less than 20 mins. To enable detection by the naked eye, we used SYBR Green I for the colorimetric detection of the amplification reaction and direct visualization via lateral flow (LF) strip.

## Materials and methods

### Sample preparation

A total of 88 RT-qPCR positive and 45 RT-qPCR negative nasopharyngeal swabs samples from a recent COVID-19 outbreak in Malaysia (2020) were collected by Hospital Sungai Buloh, Malaysia. Total RNA was extracted using QIAamp Viral RNA Minikit (Qiagen, Germany) according to the instruction manual. This study has been approved by the Medical Research Ethics Committee (MREC) Ministry of Health Malaysia (NMRR-20-535-53855). Our study used the archived anonymized samples from nasopharyngeal or oropharyngeal swabs for diagnosis of COVID-19 that were already collected from the routine clinical procedures by the managing clinicians. Patients' medical records were accessed from March 2020 to October 2020. The information was recorded by the investigator in such a manner that subjects cannot be identified directly, or through identifiers linked to the subjects. Collected data from participants were recorded in an anonymous format. All subjects cannot be identified directly or indirectly. Archival of medical records and study data will be deleted after that. Results of the molecular diagnosis will be reported back to the hospital upon request. Study findings will not be informed back to the patients. Therefore, obtaining informed consent is not required for this study according to MREC committee.

### RT-RPA

Primers were designed to target the SARS-CoV-2 nucleocapsid (N) gene (GenBank accession no MN988713.1, LC528233.1 and MT123293.1). All the primers and probe were synthesized by Sangon (Sangon Biotech, Shanghai, China) (Table 1).

**Table 1:** Oligonucleotide primers and probe for RT-RPA assay.

Name	Sequence (5’→3’)
RPA-LF Probe	5’-[FAM-dT]-TTGTTCGTTCTATGAAGACTTTTTAGAGTA[dSpacer]CATGACGTTCGTGTT-3’-[C3- spacer]
RPA-LF Forward primer	5’-TTGTTCGTTCTATGAAGACTTTTTAGAG-3’
RPA-LF Reverse primer	5’-(Biotin)-TTTGATCGCGCCCCACTGCGTTCTCCATTC -3’

**Fig 1:**
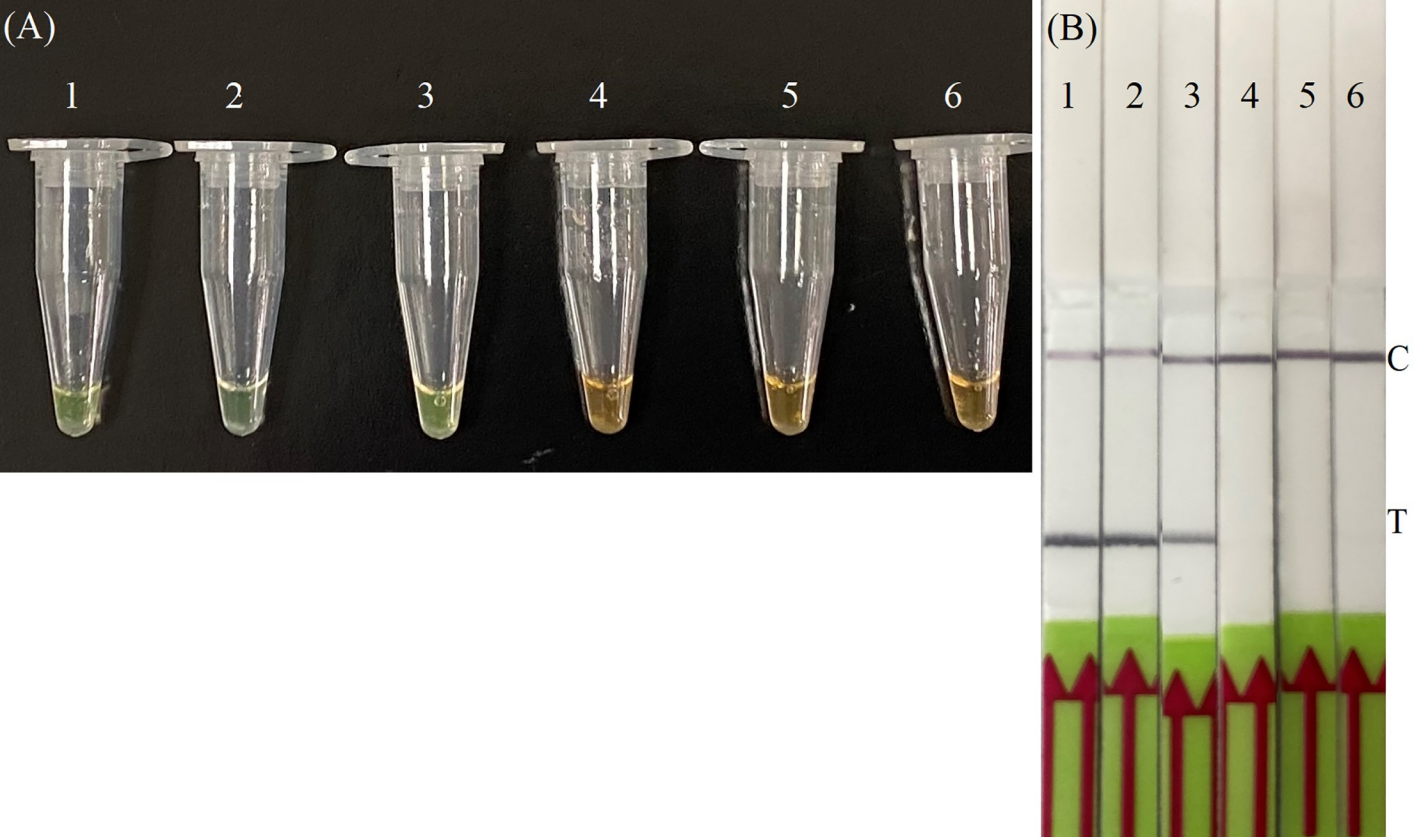
**Detection of RT-RPA amplification of SARS-CoV-2 by (A) SYBR Green I.** Representative image of specificity of RT-RPA on RNA extracted from patient samples. RT-RPA products of SARS-CoV-2 were endpoint‐detected by SYBR Green I. Tube 1: positive control, showing a bright green colour; Tubes 2 and 3: two positive samples; Tubes 4 and 5: two negative samples; Tube 6: no template control (NTC) contained water, showing light orange colour. **(B) Lateral flow strip**.

RT-RPA assay was performed according to the TwistAmp®nfo kit manufacturer's instructions (TwistDx, Cambridge, UK). The reaction mixture contained 29.5μL rehydration buffer, 5 μL extracted RNA template, 2.1 μL forward and reverse primer (10 μM) each, 0.6 μL LF probe (10 μM), 0.5 μL RNase inhibitor, 0.5 uL μL RevertAid Reverse Transcriptase (Thermo Fisher Scientific, US), 7.2 μL dH2O and 2.5 μL magnesium acetate (280 mM). The RT-RPA reaction mixtures were incubated at 42 °C for 20 mins with a brief vortexing after 2 mins. Endpoint assessment was done by visual inspection following the addition of 1 μL of 375x SYBR Green I (TaKaRa Bio, Tokyo, Japan) to the 25‐μL RPA product. To avoid opening the tube, SYBR Green I was added to the cap of the tube during the initial setup of the reaction mixture. All reactions were performed along with non-template controls. The tube was briefly centrifuged after 20 mins of incubation. A positive amplification was indicated by a colour change from light orange to bright green (Fig 1). By contrast, the solution remained light orange in the absence of RPA amplification, indicating that SARS-CoV-2 DNA was not present. The outcome of the RT-RPA was also visualized using Milenia's Genline 1-Hybridetect-2 LF strip (Milenia GmbH, Germany) duplexes labeled with anti-FAM gold conjugates and anti-Biotin antibodies for detection of RPA amplified nucleic acids. After RT-RPA, 1 μL of the RPA amplification product was diluted in 99 μL of dilution buffer (provided with the kit). LF strip was placed vertically into the diluted mixture and incubated at room temperature for < 5 mins. A positive amplification was indicated by both control and test lines visualized on the strip within 5 mins. The test was considered invalid if the control line was absent [4].

The positive amplification product was indicated by both test line (T) and control line (C) on the strip visualized simultaneously within 5 mins. A sample was interpreted as negative if only (C) was visible. The test was considered invalid if the control line was absent. Strip 1 = Positive control; Strips 2 and 3 = two positive samples; Strip 4: NTC; Strip 5 and 6: two negative samples.

### RT-qPCR

The RT-qPCR assay was performed on Bio-Rad CFX qPCR (Bio-Rad, USA) as previously described [5]. The reactions were prepared as a 25 μL reaction volume containing 12.5 μL 2x reaction mix, 0.5 μL enzyme mix and 5 μL extracted RNA.

### Analytical sensitivity and specificity

To determine the analytical sensitivity of the SARS-CoV-2 RT-RPA assay, *in vitro* transcript RNAs were prepared using previously published method [9]. Briefly, target gene fragments were cloned to pGEM-T vector as described in the manufacturer’s protocol (Promega, USA). Then, the recombinant plasmid was linearized downstream of the targeting segments with restriction endonuclease. *In vitro* transcribed RNAs were prepared with RiboMAX™ System (Promega, USA) and digested by deoxyribonuclease (DNase) I and purified by phenol-chloroform extraction method. The limit of detection (LOD) was determined using 10-fold serially diluted *in vitro* transcript RNA with known numbers of nucleic acid copies (10 cp/μL, 5 cp/μL, 2 cp/μL, 1 cp/μL and 0.1 cp/μL) and comparing the assay with RT-qPCR. One uL of template was used in the RT-RPA and RT-qPCR assays [6]. The analytical sensitivity assays were repeated 5 times to allow probit regression analysis to accurately determine the limit of detection of RT-RPA.

The specificity of the RT-RPA assay was determined by using genomic RNA of coronaviruses (HCoV-OC43 and SARS-CoV), adenovirus, human metapneumovirus, influenza A (A/H1pdm2009 and A/H3) viruses, influenza B virus, parainfluenza virus 3, rhinovirus A, respiratory syncytial virus B and enterovirus D68. However, we did not include Coronavirus 229E555, Coronavirus NL63, Coronavirus HKU1 and MERS due to shipping restriction of these items to our country at this moment.

### Clinical sensitivity and specificity

Ten RT-qPCR positives and 10 negatives were used for initial optimization of the RT-RPA assay. The remainder of 78 RT-qPCR positive and 35 negative RNA samples were used for blind testing. Clinical sensitivity was calculated as (number of true positives)/(number of true positives + number of false negatives) and clinical specificity was calculated as (number of true negatives)/(number of true negatives + number of false positives) compared to RT-qPCR (Table 2).

**Table 2:** Clinical sensitivity, specificity, positive predictive value, and negative predictive value of the RT-qPCR and RT-RPA.

	RT-qPCR			Clinical sensitivity (%) (95% CI)	Clinical specificity (%) (95% CI)	Positive predictive value (%) (95% CI)	Negative predictive value (%) (95% CI)
RT-RPA	Positive	Negative	Total				
Positive	77	0	77	98.7 (93.1–99.9)	100 (90–100)	80.4 (36.9–96.6)	98.8 (94.6–99.9)
Negative	1	35	36				
**Total**	**78**	**35**	**113**				

RT-RPA: reverse transcription-recombinase polymerase amplification; RT-qPCR: reverse transcription-qualitative polymerase chain reaction

## Results

The optimal detection conditions of SARS-CoV-2 RT-RPA assay was explored with a range of temperatures (30, 37 and 45°C) and incubation time (5, 10, 15, 20, 25 and 30 mins). The results showed that the optimal temperature was 37°C. The amplification products could be detected between 15 to 20 mins using SYBR Green and/or LF strip. Colour changes and clear band were observed using SYBR Green and LF strip, respectively within 30 seconds (Fig 1). Therefore, the subsequent SARS-CoV-2 RT-RPA assay was performed in a simple heater block at 37°C for 20 mins for visual detection using SYBR Green and LF strip.

**Fig 2:**
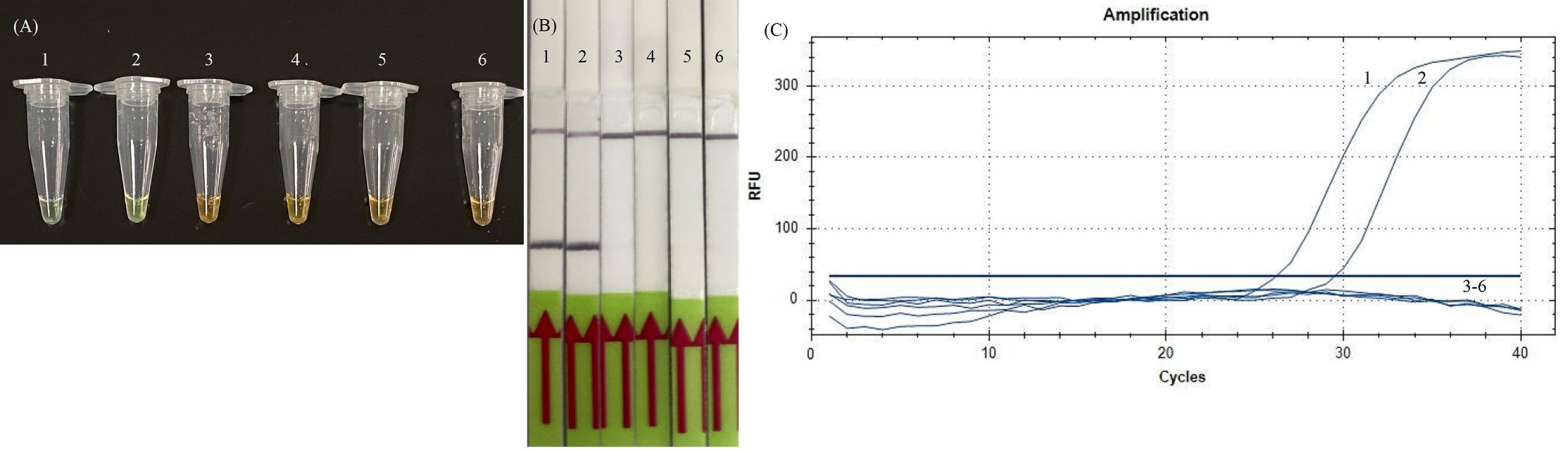
Analytical sensitivity of the RT-RPA comparing with RT-qPCR. The limit of detection (LOD) was determined using 10-fold serially diluted *in vitro* transcript RNA with known numbers of nucleic acid copies (10 cp/μL, 5 cp/μL, 2 cp/μL, 1 cp/μL and 0.1 cp/μL) (Tubes 1 to 5). Tube 6 = NTC. The detection limit of (A) RT-RPA by SYBR Green I, (B) RT-RPA by LF and (C) RT-qPCR was 5 cp/μL.

To determine the analytical sensitivity of RPA assay, we carried out the SARS-CoV-2 RT-RPA assay with the quantity of RNA template ranging from 0.1 to 20 cp/μL. The result showed that the minimum detection limit of both RT-RPA and RT-qPCR assays was 5 copies per reaction (1 μL was used in each reaction) (Fig 2). The specificity of the assay was assessed among other viral pathogens and no amplifications were observed. The limit of detection of RT-RPA at 95% probability was 7.659 cp/μL of transcript RNA. The calculation was performed by using IBM SPSS Software Statistic 23.

SARS-CoV-2 RT-RPA assay was used to screen 78 positive and 35 negative nasopharyngeal clinical samples confirmed by RT-qPCR. The results of those assays showed that a total of 77 out of 78 positive clinical specimens tested positive with RT-qPCR were detected positive while none of the negative clinical samples were detected positive (S1 Table). Therefore, the sensitivity and specificity of SARS-CoV-2 RT-RPA assay were 98.7% and 100%, respectively.

## Discussion

RT-RPA could be monitored in real time (during amplification) or end point by addition of SYBR Green I. The end product of the RT-RPA can also be detected conveniently using a LF strip. On balance, the end point detection using SYBR Green or LF strip used less instrumentation than real-time monitoring, thus reducing the overall cost of the test and suited to resource-limited settings [7]. Moreover, the test results either appear as visible bands or show a colour change that can be easily seen and interpreted with the naked eye. In all cases, the amplification process and detection were performed in less than 25 mins at 37°C with a detection limit of as low as 5 RNA copies which is equivalent to the analytical sensitivity of RT-qPCR. The specificity of the assay was assessed using other viral pathogens with similar clinical symptoms and no cross-reactions were observed. Compared to LF strip, using SYBR Green means not having to open the tube which can lead to aerosol contamination. A visible change in colour shows a clear positive/negative result. Compared to detecting via SYBR Green I, the cost of the LF-RPA assay is relatively higher.

The clinical sensitivity and specificity of SARS-CoV-2 RT-RPA assay were 98% and 100%, respectively. This is comparable to the real-time RT-RPA assays developed by Behrmann et al. (2020) [8], where 8 positive and 11 negative SARS-CoV-2 nasopharyngeal swab samples were tested with 100% sensitivity and specificity. However, an isothermal fluorescence reader was necessary to interpret the results.

Other isothermal amplification techniques such as Loop mediated isothermal amplification (LAMP) assays have been developed for rapid detection of SARS-CoV-2 [9, 10]. The RT-RPA assay developed here has two benefits compared to the RT-LAMP assay: (i) shorter run time (≤20 mins for RT-RPA versus ≥30 mins for RT-LAMP), (ii) lower temperature with lower energy consumption (37°C for RT-RPA versus 65°C for RT-LAMP). These features allow RPA to be more suitable for onsite testing or rapid diagnosis in laboratories which are not well equipped. In one report, RT-RPA assay was able to amplify patient RNA samples by using heat generated in a closed fist for the detection of foot-and-mouth disease virus within 17 mins [11]. RPA assay has been used in a mobile laboratory in combination with a commercial magnetic bead-based DNA/RNA extraction kit for point-of-care rapid diagnosis of dengue infection [12].

In a separate study, a novel assay combining RPA and LAMP techniques in a single tube has also been developed for the detection of SARS-CoV-2. This assay has greater sensitivity than LAMP alone and the whole process can be completed within 1 hour. However, this assay never tested actual clinical samples [13].

Behrmann et al. (2020) detected the RT-RPA end product by real time PCR machine. The LOD of the RT-RPA was 7 cp/μL which is comparable to our study (7.659 cp/μL). While in our study, the RT-RPA end product was detected by observing the color changes and lateral flow strip which omitted the need of a real-time PCR machine. This would save on assay costs and facilitate onsite diagnosis in the field [8].

Kim et al. (2020) managed to detect 4 copies per 50 μL reaction within 10 min, or 8 copies within 8 min using RPA. However, their protocol requires tedious steps. The amplified product needs to be digested to single stranded RPA product (SSRPA) before loading onto the lateral flow for final detection. This method is costlier as additional enzyme is need for digestion and higher chances for cross contamination occurred [14].

As isothermal assay is highly sensitive. Precautions such as preparing the master mix in a separate room and frequent changing of gloves should be taken. Furthermore, addition of uracil-DNA-glycosylase-supplemented may help to eliminate carryover contamination as demonstrated in PCR and LAMP assays [15, 16].

## Conclusions

SARS-CoV-2 RT-RPA assay has been successfully developed and detected using SYBR Green I and/or LF strip which offer a rapid and simple solution for field-based nucleic acid testing. This rapid- and sensitive SARS-CoV-2 RT-RPA assay can be integrated into point‐of‐care diagnosis for SARS-CoV-2 detection, especially in remote areas where laboratory resources are limited.

## Supporting information

S1 Table Real time RT-PCR and RT-RPA results. (DOCX)
