# Correction: Development of a reverse transcription recombinase polymerase amplification assay for rapid and direct visual detection of Severe Acute Respiratory Syndrome Coronavirus 2 (SARS-CoV-2)

**DOI:** 10.1371/journal.pone.0249100

**Published:** 2021-03-18

**Authors:** Yee Ling Lau, Ilyiana binti Ismail, Nur Izati binti Mustapa, Meng Yee Lai, Tuan Suhaila Tuan Soh, Afifah Haji Hassan, Kalaiarasu M. Peariasamy, Yee Leng Lee, Maria Kahar Bador Abdul Kahar, Jennifer Chong, Pik Pin Goh

The Funding statement is incorrect. The correct Funding statement is as follows: This work was supported by the Prototype Research Grant Scheme (PRGS) (PR001-2020B) from the Ministry of Higher Education, Malaysia.
