# Supplementary material for: Development of a reverse transcription recombinase polymerase amplification assay for rapid and direct visual detection of Severe Acute Respiratory Syndrome Coronavirus 2 (SARS-CoV-2)
Source: PLoS One. 2021 Jan 6;16(1):e0245164. doi: 10.1371/journal.pone.0245164 (PMC7787525; doi:10.1371/journal.pone.0245164)
Supplement: S1 Table — (DOCX) [file pone.0245164.s001.docx]

**S1 Table: Real time RT-PCR and RT-RPA results.**

| Patient Sample No. | RT-PCR | RT-RPA | Patient Sample No. | RT-PCR | RT-RPA |
| --- | --- | --- | --- | --- | --- |
| N2141 | + | + | N2540 | - | - |
| N2096 | + | + | N2448 | - | - |
| N2106 | + | + | N2556 | - | - |
| N2110 | + | + | N2564 | - | - |
| N2111 | + | + | N2509 | - | - |
| N1932 | + | + | N2517 | - | - |
| N1962 | + | + | N2525 | - | - |
| N2016 | + | + | N2533 | - | - |
| N2059 | + | + | N2451 | - | - |
| N2054 | + | + | N2557 | - | - |
| N2117 | + | + | N2565 | - | - |
| N1966 | + | + | N2526 | - | - |
| N1955 | + | + | N2534 | - | - |
| N1971 | + | + | N2197 | - | - |
| N1888 | + | + | N2550 | - | - |
| N1982 | + | + | N2566 | - | - |
| N1893 | + | + | N2519 | - | - |
| N1908 | + | + | N2527 | - | - |
| N1819 | + | + | N2535 | - | - |
| N1852 | + | + | N2543 | - | - |
| N1763 | + | + | N2164 | - | - |
| N1515 | + | + | N2165 | - | - |
| N1552 | + | + | N2166 | - | - |
| N1578 | + | + | N2198 | - | - |
| N1495 | + | + | N2168 | - | - |
| N1856 | + | + | N2169 | - | - |
| N1934 | + | + | N2171 | - | - |
| N1961 | + | + | N2173 | - | - |
| N2007 | + | + | N2174 | - | - |
| N2040 | + | + | N2175 | - | - |
| N1590 | + | + | N2177 | - | - |
| N1938 | + | + | N2178 | - | - |
| N1746 | + | + | N2199 | - | - |
| N1939 | + | + | N2181 | - | - |
| N1802 | + | + | N2182 | - | - |
| N2253 | + | + |  |  |  |
| N2516 | + | + |  |  |  |
| N2529 | + | + |  |  |  |
| N2563 | + | + |  |  |  |
| N2170 | + | + |  |  |  |
| N2176 | + | + |  |  |  |
| N2185 | + | + |  |  |  |
| N2206 | + | + |  |  |  |
| N2253 | + | + |  |  |  |
| N2259 | + | + |  |  |  |
| N2231 | + | + |  |  |  |
| N2252 | + | + |  |  |  |
| N471 | + | + |  |  |  |
| N475 | + | + |  |  |  |
| N968 | + | + |  |  |  |
| N1289 | + | + |  |  |  |
| N1357 | + | + |  |  |  |
| N604 | + | + |  |  |  |
| N24 | + | + |  |  |  |
| N1334 | + | + |  |  |  |
| N33 | + | + |  |  |  |
| N85 | + | + |  |  |  |
| N832 | + | + |  |  |  |
| N891 | + | + |  |  |  |
| N1362 | + | + |  |  |  |
| N86 | + | + |  |  |  |
| N933 | + | + |  |  |  |
| N1007 | + | + |  |  |  |
| N1013 | + | + |  |  |  |
| N451 | + | + |  |  |  |
| N898 | + | + |  |  |  |
| N452 | + | + |  |  |  |
| N477 | + | + |  |  |  |
| N813 | + | + |  |  |  |
| N1289 | + | + |  |  |  |
| N1223 | + | + |  |  |  |
| N1244 | + | + |  |  |  |
| N1523 | + | + |  |  |  |
| N1521 | + | + |  |  |  |
| N1308 | + | + |  |  |  |
| N1506 | + | + |  |  |  |
| N180 | + | + |  |  |  |
| N455 | + | - |  |  |  |
